# Home care nurses’ management of high-risk medications: a cross-sectional study

**DOI:** 10.1186/s40545-022-00476-2

**Published:** 2022-11-21

**Authors:** Irina Dumitrescu, Minne Casteels, Kristel De Vliegher, Laura Mortelmans, Tinne Dilles

**Affiliations:** 1 Faculty of Medicine and Health Sciences, Department of Nursing Science and Midwifery, Centre For Research and Innovation in Care (CRIC), Nurse and Pharmaceutical Care (NuPhaC), University of Antwerp, Antwerp, Belgium; 2 Nursing Department, White-Yellow Cross of Flanders, Brussels, Belgium; 3 Department of Pharmaceutical and Pharmacological Sciences, KU Leuven, Leuven, Belgium

**Keywords:** Community care, Cross-sectional study, High-risk medications, Home care nurse, Medications care

## Abstract

**Background:**

High-risk medications use at home entails an increased risk of significant harm to the patient. While interventions and strategies to improve medications care have been implemented in hospitals, it remains unclear how this type of medications care is provided in the home care setting. The objective was to describe home care nurses’ management of high-risk medications.

**Methods:**

A cross-sectional, descriptive design was set up in home care nurses in Flanders, Belgium. Participants were recruited through convenience sampling and could be included in the study if they provided medications care and worked as a home care nurses. Participants completed an online structured questionnaire. Questions were asked about demographic information, work experience, nurses’ general attitude regarding high-risk medications, contact with high-risk medications and the assessment of risk and severity of harm, specific initiatives undertaken to improve high-risk medications care and the use of additional measures when dealing with high-risk medications. Descriptive statistics were used.

**Results:**

A total of 2283 home care nurses participated in this study. In our study, 98% of the nurses reported dealing high-risk medications. Home care nurses dealt the most with anticoagulants (96%), insulin (94%) and hypnotics and sedatives (87%). Most nurses took additional measures with high-risk medications in less than 25% of the cases, with the individual double check being the most performed measure for all high-risk medications except lithium. Nurses employed by an organization received support mostly in the form of a procedure while self-employed nurses mostly look for support through external organizations and information sources.

**Conclusions:**

The study shows several gaps regarding high-risk medications care, which can imply safety risks. Implementation and evaluation of more standardized high-risk medications care, developing and implementing procedures or guidelines and providing continuous training for home care nurses are advised.

**Supplementary Information:**

The online version contains supplementary material available at 10.1186/s40545-022-00476-2.

## Background

Home care nurses are considered crucial in the care for patients who want to stay at home for as long as possible. The dynamic characteristic of this setting is related to specific patient profiles and activities depending on the acute or long-term modalities of the home care provision [1, 2]. Specific competencies have been appropriated to nurses, such as being able to act as autonomous professionals; form interpersonal relationships with patients and family members, to collaborate inter- and multiprofessionally, to possess technical and scientific knowledge of the specific field, and to be able to coordinate and delegate care [1]. Nurses are considered experts in clinical and technical actions, such as the provision of safe medications care [2]. These actions include preparing, verifying and administering medications, updating their knowledge on medications, monitoring the therapy efficacy, observing adverse reactions and educating patients on their medications [3–5]. Literature suggests that nurses indeed draw on knowledge and clinical reasoning when administering medications safely, after assessing the patient and his/her medications [6]. It is considered a nurse’s responsibility to regularly update his/her knowledge, skills and clinical practices related to medications care [7].

Recently, more attention has been given to high-risk medications. Specifically for the home care setting, high-risk medications are considered “*medications with an increased risk of significant harm to the patient where the consequences of this harm can be more serious than those with other medications*” [8]. In a recent Delphi study, a multidisciplinary panel of experts found that specific attention is considered necessary for the following set of high-risk medications in community care, in order to reduce adverse drug events: digoxin, antiarrhythmics, methotrexate, hypnotics and sedatives, immunosuppressants, dual platelet therapy, insulin, antipsychotics, carbamazepine, lithium, anticoagulants, oral hypoglycaemic drugs, phenytoin, opioids and chemotherapeutic drugs [9].

The increase in this type of drug use at home inherently entails the increased risk of adverse drug events with significant harm to the patient [10]. It has been recognized that careful medications monitoring by a nurse can lead to a decrease of the impact of adverse medications effects, promoting patient safety [2, 11]. This has led to several interventions and strategies implemented in hospitals and long-term care facilities to improve high-risk medications care, such as targeted medications reviews and medications simplifications, the use of risk-assessment tools, the process of deprescribing high-risk medications, the implementation of quality and safety monitoring systems, multidimensional interventions with aspects of discharge planning and patient education and continuous education programmes [12–15].

As research is primarily focused on the hospital setting, it remains unclear how nurses manage high-risk medications care in a home setting, what specific attention is given to this type of medications and how home care nurses feel when they’re confronted with this type of medications. Therefore, this study aimed to describe the home care nurses’ management of high-risk medications.

What is already known about the topic?

- High-risk medications are medications with an increased risk of significant harm to the patient.
  Careful medications monitoring by a nurse can lead to a decrease of the impact of adverse medications effects, promoting patient safety. What this paper adds
- Nurses take additional measures when dealing with high-risk medications but in an inconsistent way, with the individual double check being the most performed measure for all high-risk medications except lithium.
- Home care nurses feel they have sufficient knowledge and competences regarding high-risk medications, but they are aware of the risks that high-risk medications pose and feel the need of extra training in this matter.
- Home care nurses employed by an organization receive support mostly in the form of a high-risk medications procedure and training while self-employed nurses mostly look for training and checklists about high-risk medications.

## Methods

### Aim

To describe the home care nurses’ management of high-risk medications: the extent to which home care nurses work with high-risk medications, how they manage these medications and how home care nurses are supported in dealing with high-risk medications.

### Study design, setting and participants

A quantitative cross-sectional observational study was performed in Flanders, Belgium, with both home care nurses in employment and self-employed home care nurses providing care to patients living at home. Home care organizations and self-employed nurses were contacted to participate in the study and recruit home care nurses for the study. Information about the study was provided through e-mail, telephone and directly during (personal) meetings. Reminders were regularly sent to increase the response rate. Participants were recruited through convenience sampling and could be included in the study if they worked as a home care nurses and provided medications care.

### Data collection

The questionnaire for this study consisted of 3 parts and was developed based on results from previous studies of the HaRMonIC project and literature. The first part of the questionnaire included closed-ended questions (age, gender, level of education, employment type, work experience (factors) in home care and employment regimen). Work pressure, work pleasure, opportunities for training, and involvement in optimizing therapeutic effects preventing adverse events were to be scored on a 10-point Likert scale.

The second part concerned the general home care nurses’ management of high-risk medications. Statements on general attitude regarding high-risk medications (5-point Likert scale, 1 = never, 5 = always) and closed-ended questions were asked about specific initiatives undertaken to improve high-risk medications care, such as the use of a list of high-risk medications, reminders, training, procedures or checklists. The use of additional measures when dealing with high-risk medications was questioned through a 5-point Likert scale (1 = never, 5 = in more than 75% of the cases). Additional measures were defined as measures that were not usually taken when dealing with other medications. Nurses were also asked to estimate how many times during a shift they dealt with high-risk medications in general. This was defined as preparing high-risk medications, administering high-risk medications, monitoring a patient, etc.

The third part was related more specifically to the 15 high-risk medications aforementioned [8]. For each high-risk medications, the home care nurses indicated how often they provided care related to that drug during a fulltime working week and which additional measures were taken. Home care nurses could choose from a list of possible measures, based on the results of a Delphi study (2021) and on the package leaflet of the products. For all 15 high-risk medications, a general set of 12 measures was presented (see Additional file 1 for the entire list). For each specific high-risk medications additional measures were added to this list, if they applied to the high-risk medications (e.g. monitoring risk of falls in the case of anticoagulants).

Finally, the nurses were asked to assess the risk of harm following the use of the specific high-risk medications and the severity of this harm through a 10-point scale.

In order to limit the length and complexity of the questionnaire and to achieve satisfactory response rates, the high-risk medications in the third part of the questionnaire were randomly assigned to 3 groups of 5 high-risk medications each. Each nurse was therefore presented with a set of questions regarding only 5 high-risk medications. The first group consisted of digoxin, antiarrhythmics, methotrexate, hypnotics and sedatives and immunosuppressants. The second group consisted of dual platelet therapy, insulin, antipsychotics, carbamazepine and lithium. The third group consisted of anticoagulants, oral hypoglycaemic drugs, phenytoin, opioids and chemotherapeutic drugs. The questionnaires were randomly allocated to the participants by the survey software.

The questionnaire was piloted by several graduate degree nurse students, checking the questionnaire for understandability, feasibility, and the time to fill in the questionnaire. The questionnaire was then introduced in Snap Survey Software, allowing online data collection. A link to the questionnaire was distributed directly to participating self-employed nurses and to home care organizations, asking further distribution to their home care nurses. Questionnaires could be filled in between December 2018 and March 2019.

### Statistical analysis

Data were exported from Snap Survey to IBM SPSS Statistics 27 for analysis. In line with the descriptive nature of the research questions and the design, mainly descriptive statistics were used. Results are presented using percentages, means and standard deviations. Pearson Chi-square test, Kruskal–Wallis and one-way ANOVA were used to assess intergroup differences. A *p*-value < 0.05 was considered statistically significant. In order to examine if the 3 groups of 5 high-risk medications were comparable and the results could be interpreted as a combined result, intergroup differences were computed. A statistically non-significant result (*p* ≥ 0.05) here indicated that the groups are comparable and the results could be presented in a combined way.

## Results

### Description of participants

In total, 2283 home care nurses participated in the study with a mean age of 41 years (SD = 11.4). Characteristics of the participants, divided according to the group they were randomly assigned to, are provided in Table 1. Overall, most nurses were women (93%), had a diploma in nursing (58%) and were employed by a home care organization (90%). The home care nurses had an average of 14 years of experience in home care. Most nurses worked fulltime (31%) while others’ employment ranged between 20 and 80%. The nurses rated a rather high level of work pressure (7.36/10, SD = 1.5), but they take much pleasure in working in home care (8.96/10, SD = 1.1). Their willingness to engage in training outside working hours scored 6.54/10 (SD = 2.5), and they also felt they have an important role in improving the desired effects and preventing adverse drug events of medications (7.83/10, SD = 1.8).

**Table 1:** Description of participants’ characteristics (*n* = 2283)

		Group 1 (*n* = 768)^c^	Group 2 (*n* = 748)^c^	Group 3 (*n* = 767)^c^	Total (*n* = 2283)	*p-value*
Age, in years [mean (SD)]		41 (11.3)	41 (11.6)	41 (11.3)	41 (11.4)	0.797
Gender (%)	Female	92%	95%	92%	93%	0.079
	Male	8%	5%	8%	7%	
	Other	0.3%	0.1%	0%	0.1%	
Level of education (%)^a^	Diploma level (EQF level 5)	60%	60%	53%	58%	0.009
	Bachelor’s degree (EQF level 6) or higher	40%	40%	47%	42%	
Employment type (%)	Organization	92%	90%	88%	90%	0.062
	Self-employed	8%	10%	12%	10%	
Work experience in home care, in years [mean (SD)]		14 (11.6)	14 (11.8)	14 (11.6)	14 (11.7)	0.944
Employment regimen (%)	100%	31%	29%	31%	31%	0.7
	80–99%	23%	22%	22%	22%	
	50–79%	21%	21%	20%	21%	
	≤ 50%	23%	24%	23%	23%	
	Other	2%	4%	4%	3%	
Work experience factors [mean (SD)]	Work pressure^b^	7.33 (1.5)	7.47 (1.5)	7.28 (1.5)	7.36 (1.5)	0.033
	Pleasure of working in home care^b^	9 (1)	8.96 (1)	8.93 (1.2)	8.96 (1.1)	0.532
	Training outside working hours^b^	6.55 (2.5)	6.54 (2.6)	6.54 (2.5)	6.54 (2.5)	0.934
	Role in improving desired effects and prevent adverse drug events in medications therapy^b^	7.86 (1.8)	7.73 (1.8)	7.88 (1.7)	7.83 (1.8)	0.159

^a^European Qualification Framework (EQF): European Centre for the Development of Vocational Training. 2021 [Available from: https://www.cedefop.europa.eu/en/events-and-projects/projects/european-qualifications-framework-eqf.]

^b^Items scored on a 10-point scale

^c^Group 1 was questioned about digoxin, antiarrhythmics, methotrexate, hypnotics and sedatives and immunosuppressants. Group 2 was questioned about dual platelet therapy, insulin, antipsychotics, carbamazepine and lithium. Group 3 was questioned about anticoagulants, oral hypoglycaemic drugs, phenytoin, opioids and chemotherapeutic drugs. The 3 groups are presented in the same table as they were questioned in the same manner about the same subject

### Home care nurses’ general management of high-risk medications

#### Readiness regarding high-risk medications care

**Fig. 1:**
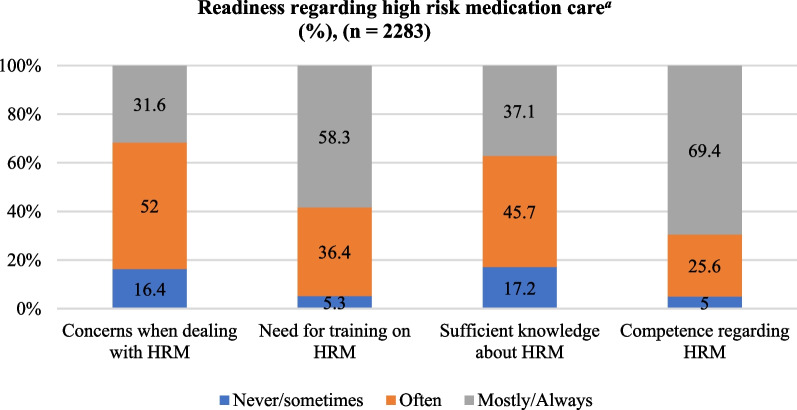
Readiness regarding high-risk medications care^a^ (%), (*n* = 2283). ^a^These 4 items were scored on a 5-point Likert scale with scores ranging from (1) “never” to (5) “always”. Scores (1) “never” and (2) “sometimes” were combined and scores (4) “mostly” and (5) “always were combined; HRM =  high-risk medications

Approximately one-third of the home care nurses reported being concerned most of the time or always when dealing with high-risk medications. More than half of the nurses feel the need for extra training with regard to high-risk medications. Almost 40% of home care nurses reported having sufficient knowledge about high-risk medications, whereas the majority feels competent when dealing with high-risk medications. The frequencies of the nurses’ readiness scores regarding high-risk medications care are presented in Fig. 1.

### (Organizational) support when dealing with high-risk medications

When dealing with high-risk medications, nurses employed by an organization received support mostly in the form of a procedure about high-risk medications (53%) and training (51%). Self-employed nurses mostly referred to training (30%) and checklists about high-risk medications (23%) provided through external organizations and information sources. Reminders about high-risk medications were the least provided type of support (24% for home care nurses in an organization and 15% for self-employed nurses). When looking at the actual use of support that was provided, numbers are high (range 68–89%). Table 2 presents the types of support provided when dealing with high-risk medications and its use.

**Table 2:** (Organizational) support, divided according to employment type (*n* = 2283)*

	(Organizational) support available		(Organizational) support was actually used, if available	
	Organization (%)	Self-employed (%)	Organization (%)	Self-employed (%)
List	36	17	69	68
Procedure	53	19	78	84
Training	51	30	86	83
Checklist	27	23	74	80
Reminders	24	15	80	89

*Support in an organization is provided through the organization, while self-employed nurses look for support through external organizations and information sources

### Additional measures when dealing with high-risk medications

When dealing with high-risk medications in general, home care nurses took additional measures in a fragmented way: 26% of the nurses took additional measures in less than 25% of the cases, whereas 23% took measures in more than 75% of the cases. Surprisingly, 15% of the home care nurses reported never taking additional measures when dealing with high-risk medications. The extent to which nurses took additional measures is spread more or less evenly across the amount of contacts with high-risk medications, meaning that there is no greater use of measures in the case of higher high-risk medications contact. Table 3 presents the frequency of additional measures taken by home care nurses when dealing with high-risk medications.

**Table 3:** Frequency of additional measures when dealing with high-risk medications (*n* = 2243)

Frequency of additional measures when dealing with high-risk medications	Nurses Total *(%)*
Never	15
In less than 25%	26
Between 26 and 50%	18
Between 51 and 75%	18
In more than 75%	23

### Home care nurses’ management of specific high-risk medications

The contact with specific high-risk medications and the use of additional measures when dealing with specific high-risk medications presented with a statistically significant difference between the 3 groups. As participant characteristics are similar in the three groups, the assignment to a group of high-risk medications had an impact on home care nurses’ contact with high-risk medications and the measures taken. Therefore, the results are reported separately for each of the 3 groups.

### Contact with high-risk medications

**Fig. 2:**
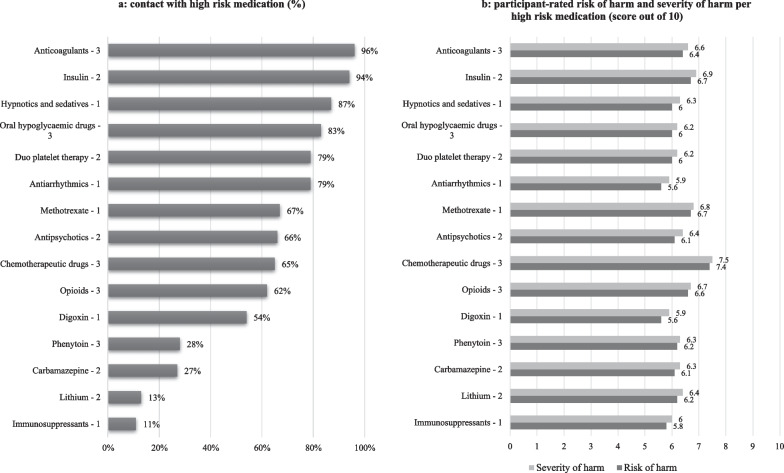
**a** Contact with high-risk medications (%). **b** Participant-rated risk of harm and severity of harm per high-risk medications (score out of 10). ^1^High-Risk Medications from group 1 (*n* = 768); ^2^from group 2 (*n* = 748); ^3^from group 3 (*n* = 767); reported only by nurses who reported contacts with the high-risk medications

In our study, 98% of the home care nurses dealt with at least one of the surveyed high-risk medications. Nurses dealt the most with anticoagulants (96%), insulin (94%) and hypnotics and sedatives (87%). Chemotherapeutics were considered having the highest risk of harm (7.4/10) and severity of harm (7.5/10), whereas both antiarrhythmics and digoxin the lowest risk of harm (5.6/10) and severity of harm (5.9/10). Figure 2 presents all surveyed high-risk medications’ contact frequencies and the scores of estimated risk of harm and severity of harm, respectively.

### Additional measures when dealing with specific high-risk medications

Of all 12 measures, the individual double check by the home care nurses themselves was performed the most (range 39.6–73.7%) for all high-risk medications except lithium. The high-risk medications where the individual double check was performed by most nurses, are methotrexate for group 1 (57.3%), insulin for group 2 (73.7%) and chemotherapeutics for group 3 (71%). In the case of lithium, nurses mostly noted important points in the nursing file (46.3%). An extra visit from the nurse is the least performed measure (range 0.6–5.4%) for all high-risk medications except insulin. The high-risk medications where an extra visit by a home care nurse was performed the least, are methotrexate for group 1 (0.6%), dual antiplatelet therapy for group 2 (1.5%) and anticoagulants for group 3 (1.8%). In the case of insulin, the least performed measure is receiving help from a colleague (8.1%).

More specific interventions, only performed when dealing with unique high-risk medications, are presented in Additional file 1. Additional file 1 shows the frequency of each additional intervention that was performed when dealing with a high-risk medications.

## Discussion

We conducted a descriptive cross‐sectional survey to investigate home care nurses’ management of high-risk medications in a home setting, the specific attention that is given to this type of medications and how the nurses feel when they are confronted with this type of medications. A large sample of home care nurses participated in this study with the majority of nurses being employed by an organization.

We conclude that most home care nurses indeed take measures when performing high-risk medications care, but in an inconsistent way. Also, 15% of nurses still does not take any additional measures in case of high-risk medications. Patient treatments may vary between providers and even within the same provider. A lack of standardization of care raises concerns regarding quality of care and patient safety, while providing standardized care could reduce variation in treatment and patient outcomes, improving the safety and quality of care [16, 17]. Barriers to clinical guideline adherence may consist of a lack of awareness or familiarity of the recommendations, not agreeing with guidelines, external barriers that limit the performance of recommended behaviour, the lack of appropriate equipment and electronic systems or the absence of a standardized care process [18, 19]. Our study did not investigate the reasons for the inconsistency in high-risk medications care by the home care nurses, but the reported lack of a clear practice guideline or procedure could be the cause. After all, only half of the nurses employed by an organization reported the presence of a procedure, and only 20% of self-employed nurses had a procedure at their disposal. Developing and implementing a high-risk medications’ guideline or policy could standardize this type of medications care and increase patient safety and quality of care. In addition, it is imperative for future researchers to identify and tackle the impeding factors for nurses to adhere to the guideline or policy.

The individual double medications check is the most performed measure by a home care nurses for nearly all high-risk medications included in the study. This standard practice of double medications check originates from the hospital setting, which implies two individuals verifying the same information. The rationale behind this practice is that two independent people are less likely to make the same mistake [20]. This standard practice has been translated to an individual double check according to the specific nature of home care, where a single nurse provides care to a number of patients generally without the presence of a colleague. The second check is therefore performed by the same single nurse, but at a different time during the patient care. In other words, the double medications check happens on an individual basis, or one could say that the single medications check is done twice. A recent systematic review evaluated the effectiveness of double checking medications and it remains unclear whether double checking is effective in reducing medications errors and improving patients outcomes [21]. Nevertheless, the Institute for Safe Medications Practices believes that double medications checks are important in medications care and should be performed in a selective and proper way. Better results can even be seen from the use of automated double checks such as computerized screening and barcode scanning [22]. Moreover, it is unclear whether the individual double medications check (or the double single medications check), as performed by the nurses in our study, is effective in reducing medications errors as the main criterion of the independent check is not met. Although the individual double medications check is a very different concept, research on its effectiveness would be useful as high-risk medications care continues to be shifted to the primary care setting. It is important to consider whether the strategies that are currently being used in this setting are in fact effective and if not, that alternatives are offered.

Our study revealed that home care nurses employed by an organization receive support mostly in the form of a procedure about high-risk medications while self-employed nurses mostly look for support through external organizations and information sources about high-risk medications, but the extent to which this support is provided remains low. Human medical errors can indeed be reduced by the introduction of operational procedures, which reduce unnecessary complexity and provide systems for controllability and monitoring [23]. Clinical guidelines also reduce variation in practice and facilitate evidence-based practice and are therefore an apt choice for improving patient health outcomes, clinical decisions and the quality and efficiency of care [24]. Developing and implementing such procedures are therefore strongly recommended. Attention should however be given to the careful and successful implementation of new procedures, as the introduction of new procedures or systems in itself can lead to medical errors and can be the direct cause of accidents [25]. In addition, the recommendations in the procedure or guideline should be carefully and deliberately included, as they may be inaccurate or rigid and inflexible [24].

The results regarding the high-risk medications awareness appear contradictory at first sight. One third of the home care nurses in our study reports being concerned all or most of the time when dealing with high-risk medications, but at the same time they report having sufficient knowledge about high-risk medications. In addition, the majority of nurses feels competent when dealing with high-risk medications, yet more than half of the nurses feels the need for extra training with regard to high-risk medications. When analysing this result, the majority of nurses clearly indicates having enough or sufficient knowledge and being competent regarding high-risk medications, but they are aware of the risks that high-risk medications pose and feel the need of extra training in this matter. A recent systematic review highlighted the importance of nurses’ continuing professional development. By updating their knowledge and skills, nurses can improve and maintain their competences and thus the quality of care they provide [26]. Besides, engaging in continuous training reduces work-related stress among nurses [27]. It has been suggested that nurses’ motivation for continuous training may differ according to their work experience. Younger nurses are willing to expand their skills and develop professionally and assume other roles, while older nurses report reaching a high level of expertise and skills, due to a significant daily experience, and the need to refine their skills is only limited [26]. Literature also suggests that nurses rely on knowledge and clinical reasoning when administering medications safely, after assessing the patient and his/her medications [6]. It’s a nurse’s responsibility to regularly update his/her knowledge, skills and clinical practices related to medications care. Medications management is addressed in nurse education, but curricula tend to fall short as they primarily focus on aspects necessary for correct administration of medications. In order to reduce medications errors, the continuous education of nurses should be taken into account [7]. It can therefore be recommended to prioritize this objective and develop specific training strategies for home care nurses, with attention to different phases in the learning process.

### Limitations

The biggest limitation regarding our study is the fact that despite the similarity of respondents in each of the 3 groups of home care nurses, there were statistically significant differences in the contact with high-risk medications and measures taken by nurses in each of the three groups. This suggests that the allocation of high-risk medications to each of the three groups resulted in groups that were not comparable. Comparison of actions of nurses between different groups of high-risk medications was therefore not performed.

The majority of the home care nurses that participated in the study were employed by an organization. It is possible that there is an overrepresentation in the results as far as their opinion is concerned and results cannot be generalized for the whole setting of home care nursing.

One of the comments we received during the study is that several nurses did not participate due to feelings of uncertainty because of the confrontational nature of the questionnaire and lack of knowledge of the high-risk medications. This may have led to an overestimation of the reported degree of competence with regard to high-risk medications care as nurses with knowledge in the matter participated. In addition, it is also possible that the nurses answered desirably to the questions regarding the measures taken.

All home care nurses were presented with a list of possible measures that can be performed when dealing with high-risk medications. This list was based on previous research and pharmaceutical insight. We cannot guarantee that the list is exhaustive as certain effective measures may be omitted.

## Conclusion

To our knowledge, this is the first study describing the management of home care nurses when dealing with high-risk medications care. Home care nurses indeed take measures when performing high-risk medications care, but in an inconsistent way. Home care nurses feel they have sufficient knowledge and are competent regarding high-risk medications, but they are aware of the risks that high-risk medications pose and feel the need of extra training in this matter. Standardizing care, developing and implementing procedures or guidelines and providing continuous training depending on the amount of work experience can improve high-risk medications care provided by home care nurses. However, more research is needed to investigate which interventions and strategies could indeed improve high-risk medications care.

## Supplementary Information

**Additional file 1: Table 1:** % of nurses taking additional safety measures. **Table 2:** frequency of (general) additional measures performed by HCN when dealing with HRM.

## Data Availability

Not applicable.
