# Supplementary material for: Home care nurses’ management of high-risk medications: a cross-sectional study
Source: J Pharm Policy Pract. 2022 Nov 21;15:88. doi: 10.1186/s40545-022-00476-2 (PMC9682630; doi:10.1186/s40545-022-00476-2)
Supplement: Supplementary file 1 — Additional file 1: Table 1: % of nurses taking additional safety measures. Table 2: frequency of (general) additional measures performed by HCN when dealing with HRM. [file 40545_2022_476_MOESM1_ESM.docx]

Home care nurses’ management of high-risk medications: a cross-sectional study

| **Supplementary table 1: % of nurses taking additional safety measures** | | |
| --- | --- | --- |
| Anticoagulants (n=737) ***** | Risk of falls monitoring | 65% |
|  | ADR monitoring | 57% |
|  | Patient education | 28% |
| Insulin (n=704)***** | Measure glycaemia | 78% |
|  | Adjusting dose according to glycaemia | 65% |
| Hypnotics and sedatives (n=665)***** | ADR monitoring | 44% |
|  | Patient education | 41% |
| Oral hypoglycaemic drugs (n=636)***** | Measure glycaemia | 54% |
|  | Patient education | 52% |
| Dual platelet therapy (n=588)***** | Hematoma monitoring | 62% |
|  | Gastric bleed monitoring | 45% |
|  | Patient education | 23% |
| Antiarrhythmics (n=604)***** | Toxic symptoms monitoring | 34% |
|  | Parameter monitoring | 33% |
|  | Patient education | 11% |
| Methotrexate (n=517)***** | Correct waste removal | 69% |
|  | Administration frequency control | 32% |
| Antipsychotics (n=492)***** | Monitoring signs of metabolism | 27% |
|  | Patient education | 25% |
|  | Parkinsonism monitoring | 24% |
| Chemotherapeutic drugs (n=500)***** | ADR monitoring | 63% |
|  | Correct waste removal | 68% |
|  | Patient education | 58% |
|  | No breaking of pills | 54% |
| Opioids (n=473)***** | ADR monitoring | 65% |
| Digoxin (n=414)***** | Toxic symptoms monitoring | 31% |
|  | Parameter monitoring | 30% |
|  | Patient education | 20% |
| Phenytoin (n=214)***** | Patient education | 31% |
| Carbamazepine (n=205)***** | Patient education | 14% |
| Lithium (n=95) | Toxic symptoms monitoring | 43% |
|  | Patient education | 41% |
|  | Symptoms of lower thyroid function monitoring | 27% |
| Immunosuppressants  (n=87) | Correct waste removal | 33% |
|  | Skin control malignancies | 24% |
|  | Protective measures | 23% |

* Reported only by those HCN that indicated dealing with HRM

**Supplementary table 2: frequency of (general) additional measures performed by HCN when dealing with HRM**

|  |  | Chemotherapeutic drugs (n=500) | Opioids (n=473) | Insulin (n=704) | Anticoagulants (n=737) | Phenytoin (n=214) | Antipsychotics (n=492) | Oral hypoglycaemic drugs (n=636) | Carbamazepine (n=205) | Methotrexate (n=517) | Dual platelet therapy (n=588) | Lithium (n=95) | Digoxin (n=414) | Hypnotics and sedatives (n=665) | Immuno-suppressants (n=87) | Antiarrhythmics (n=604) |
| --- | --- | --- | --- | --- | --- | --- | --- | --- | --- | --- | --- | --- | --- | --- | --- | --- |
| 1 | Individual double check | 71% | 57% | 74% | 63% | 58% | 56% | 54% | 50% | 57% | 50% | 45% | 41% | 44% | 49% | 40% |
| 2 | Important point in nurse file | 65% | 52% | 60% | 57% | 52% | 50% | 47% | 37% | 55% | 42% | 46% | 36% | 31% | 37% | 32% |
| 3 | Double check with patient | 47% | 32% | 46% | 43% | 32% | 32% | 34% | 25% | 37% | 30% | 25% | 30% | 29% | 26% | 29% |
| 4 | Administer medication self instead of patient | 48% | 41% | 49% | 36% | 41% | 42% | 30% | 32% | 41% | 27% | 27% | 20% | 22% | 26% | 18% |
| 5 | Medication out of reach of patient | 33% | 29% | 18% | 35% | 29% | 41% | 22% | 28% | 16% | 29% | 34% | 26% | 34% | 17% | 24% |
| 6 | Call GP | 33% | 25% | 39% | 40% | 25% | 32% | 27% | 28% | 11% | 26% | 30% | 21% | 15% | 21% | 21% |
| 7 | Double check with family member | 20% | 16% | 18% | 19% | 16% | 11% | 14% | 15% | 12% | 15% | 8% | 14% | 14% | 14% | 12% |
| 8 | Help from family | 21% | 21% | 19% | 14% | 21% | 14% | 16% | 17% | 9% | 12% | 15% | 12% | 14% | 9% | 10% |
| 9 | Double check with colleague | 18% | 15% | 12% | 17% | 15% | 9% | 11% | 8% | 11% | 12% | 7% | 15% | 11% | 9% | 11% |
| 10 | Staying longer with patient | 18% | 7% | 12% | 6% | 7% | 7% | 6% | 8% | 8% | 5% | 5% | 4% | 3% | 3% | 4% |
| 11 | Help from colleague | 11% | 9% | 8% | 5% | 9% | 7% | 6% | 8% | 3% | 5% | 7% | 2% | 4% | 3% | 4% |
| 12 | Extra visit | 6% | 1% | 9% | 2% | 1% | 3% | 4% | 5% | 1% | 2% | 3% | 2% | 1% | 3% | 2% |

**Legend:**

| 0-20 % |
| --- |
| 21-40 % |
| 41-60 % |
| 61-80 % |
